# Rock organic carbon oxidation CO_2_ release offsets silicate weathering sink

**DOI:** 10.1038/s41586-023-06581-9

**Published:** 2023-10-04

**Authors:** Jesse R. Zondervan, Robert G. Hilton, Mathieu Dellinger, Fiona J. Clubb, Tobias Roylands, Mateja Ogrič

**Affiliations:** 1 Department of Earth Sciences, University of Oxford, Oxford, UK; 2 EDYTEM-CNRS-University Savoie Mont Blanc (USMB), Chambéry, France; 3 Department of Geography, Durham University, Durham, UK; 4 Present Address: Department of Earth Sciences, University College London, London, UK

**Keywords:** Carbon cycle, Geomorphology, Geochemistry

## Abstract

Mountain uplift and erosion have regulated the balance of carbon between Earth’s interior and atmosphere, where prior focus has been placed on the role of silicate mineral weathering in CO_2_ drawdown and its contribution to the stability of Earth’s climate in a habitable state^1–5^. However, weathering can also release CO_2_ as rock organic carbon (OC_petro_) is oxidized at the near surface^6,7^; this important geological CO_2_ flux has remained poorly constrained^3,8^. We use the trace element rhenium in combination with a spatial extrapolation model to quantify this flux across global river catchments^3,9^. We find a CO_2_ release of ${68}_{-6}^{+18}$ megatons of carbon annually from weathering of OC_petro_ in near-surface rocks, rivalling or even exceeding the CO_2_ drawdown by silicate weathering at the global scale^10^. Hotspots of CO_2_ release are found in mountain ranges with high uplift rates exposing fine-grained sedimentary rock, such as the eastern Himalayas, the Rocky Mountains and the Andes. Our results demonstrate that OC_petro_ is far from inert and causes weathering in regions to be net sources or sinks of CO_2_. This raises questions, not yet fully studied, as to how erosion and weathering drive the long-term carbon cycle and contribute to the fine balance of carbon fluxes between the atmosphere, biosphere and lithosphere^2,11^.

## Abstract (web summary)

Silicate weathering of uplifted rock depletes atmospheric CO_2_, but oxidation of revealed rock organic carbon supplies CO_2_, offsetting depletion to a degree dependent on regional geological history.

## Main

The tectonic activity that builds mountains results in the uplift and exposure of organic carbon (OC) that has been incorporated in rocks (OC_petro_) alongside silicate mineral phases. The OC_petro_ represents carbon stored in rocks that has accumulated over millions of years, previously sequestered from the atmosphere by photosynthesis and buried in sedimentary basins^12^. Indeed, sedimentary and metasedimentary lithologies presently dominate the near-surface geology of the Earth, occupying about 64% of the Earth’s surface^13^; these lithologies have OC_petro_ mass to mass concentration (denoted as [OC_petro_]) ratios of about 0.25% to more than 1.0%, whereas igneous rocks have much lower values, effectively 0%, or in the case of some marine basalts, less than 0.1% (ref. ^14^).

Denudation supplies OC_petro_ to the surface through physical and chemical weathering^3,15^; the rate varies with rock type, relief, tectonic uplift, climate and vegetation^16,17^. Previous work has revealed OC_petro_ in soils and rivers^6,18–20^ and, using data from the solid load of rivers, quantified the erosion of unweathered OC_petro_^14^ and its global flux at ${43}_{-25}^{+61}$ MtC yr^−1^ (refs. ^14,19^). However, for weathered OC_petro_, estimated global rates of OC_petro_ oxidation and CO_2_ release currently derive from carbon cycle mass balance arguments or ballpark upscaling of global river trace element fluxes^8^ and have a range of estimates from 38 MtC yr^−1^ (ref. ^21^) to 100 MtC yr^−1^ (ref. ^22^). The uncertainty of OC_petro_ oxidation fluxes is highlighted by recent work that cites a potential overall range for CO_2_ release of 0–300 MtC yr^−1^ (ref. ^23^).

To determine the role of rock weathering in the carbon cycle, we require a robust, global quantification of OC_petro_ oxidation over Earth’s surface. Here, we combine (1) a compilation of OC_petro_ oxidation proxy data from dissolved rhenium (Re) in well-studied catchments around the world, (2) new probabilistic models of global OC_petro_ stock and denudation and (3) a spatially explicit OC_petro_ oxidation model with quantified uncertainty. This approach derives a global flux by extrapolating proxy derived OC_petro_ oxidation data, while accounting for sampling bias across variables such as denudation rate and underlying geology.

## Rhenium as an OC_petro_ oxidation proxy

The exploitation of the trace element Re as a proxy to study the oxidation of OC_petro_ across landscapes^24,25^ has been underpinned by (1) the link between OC accumulation in marine sediments and organic matter being a host of Re (refs. ^26,27^); (2) the paired loss of Re and OC_petro_ during weathering of sedimentary rocks^7,25,28^; and (3) the geochemical behaviour of Re being exported as a dissolved oxyanion^29^, flushed from a near-surface, oxidative weathering zone^25^. Studies tracking the fate of carbon released from the lithosphere during OC_petro_ weathering have found it can directly enter the atmosphere as CO_2_ (refs. ^30,31^) or first dissolve as inorganic carbon in water^32^, and some can be incorporated into microbial biomass^8^.

**Fig. 1:**
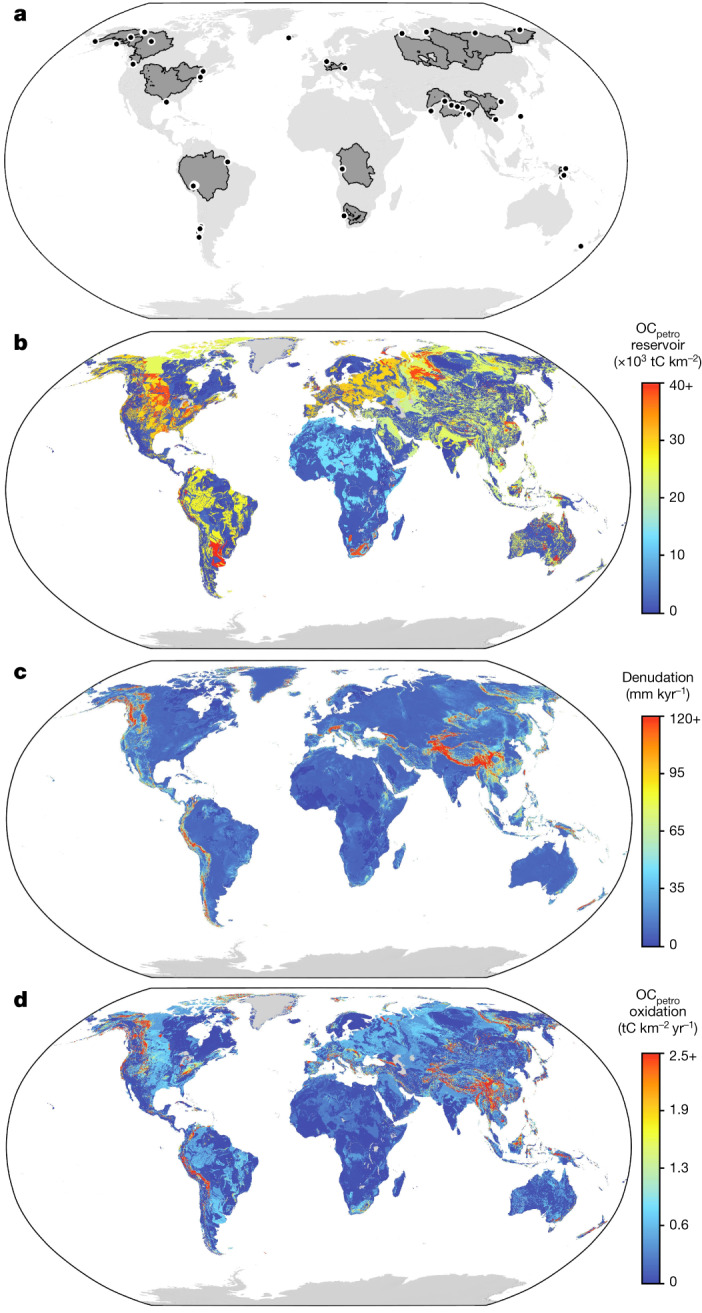
Spatial patterns of global OC_petro_ stock and oxidation. **a**, Locations of Re proxy samples and their upstream catchments. **b**, Spatially explicit estimates of OC_petro_ stocks in the upper 1 m of bedrock. **c**, Spatial model of rock denudation derived from ^10^Be data and a global raster of topographic slope. **d**, OC_petro_ oxidation fluxes extrapolated by our calibrated spatial model over the global surface.

In this study, we compile published estimates of OC_petro_ oxidation using the dissolved Re proxy, supplemented with new estimates derived from published dissolved Re concentrations^7,9,24,33^ (Methods). A forward-mixing model is used to quantify the proportion of dissolved Re from OC_petro_ oxidation using ion ratios^24,34^, while constraints on the OC_petro_ to Re ratio ([OC_petro_]/[Re]) in weathered rocks come from new and published measurements (Methods and Supplementary Table 3). Our compilation comprises 59 river basins, covering a range of drainage areas (50–5,900,000 km^2^), denudation rates and climate regimes (Fig. 1), excluding river basins with high Re pollution levels such as the Danube, Yangtze and Mississippi^9^ (Methods). The total OC_petro_ weathering flux constrained from the Re proxy across the drainage area of river basins in the dataset is 18 MtC yr^−1^ (17–23 MtC yr^−1^ within one standard deviation). The river basins in this study cover 18% of the Earth’s continental surface, and this flux would thus scale to ${98}_{-9}^{+28}$ MtC yr^−1^ globally. However, OC_petro_ stocks are spatially heterogeneous, which may affect this scaling. In the next section, we obtain a robust representative total OC_petro_ weathering flux using a spatial extrapolation model that considers patterns in OC_petro_ stock and denudation rates.

## Distribution of OC_petro_ availability

We spatially quantify the carbon stock and weathering flux of OC_petro_ at Earth’s surface using a data-driven modelling approach. Our model incorporates topographic and lithological factors to estimate OC_petro_ stocks, denudation rates and oxidative weathering rates, and is calibrated using our Re-proxy compilation (Supplementary Table 1). Unlike silicate weathering, which quickly becomes kinetically limited with increasing mineral supply by denudation^35^, OC_petro_ weathering appears to be predominately a supply-limited process^8^. This is reflected in oxidation rates which scale with erosion up to some of the highest erosion rates found on Earth, such as Taiwan and the European Alps^7,25^. Recent work at the rock outcrop scale has shown that temperature and hydrology can control OC_petro_ oxidation and CO_2_ release over time in locations with very high rates of denudation^30,31^. However, though the spatial control of denudation rates is well demonstrated on intercatchment OC_petro_ oxidation rates^7,25^ our spatial catchment-scale Re-proxy compilation does not express other environmental controls (Methods). While temperature and hydrology controls likely operate, based on the available data, their spatial predictive power is small. Here, oxidative weathering is modelled at a 1-km^2^ grid scale, resolving at the scale of catchments constrained by the Re proxy (Supplementary Table 1).

The flux of CO_2_ release by OC_petro_ oxidation, *J*_ox_ (mass × length^−2^ × time^−1^), can be expressed by a mass balance of the form:

1 $${J}_{{\rm{o}}{\rm{x}}}=\varepsilon \times \rho \times [{{\rm{O}}{\rm{C}}}_{{\rm{p}}{\rm{e}}{\rm{t}}{\rm{r}}{\rm{o}}}]\times \chi $$

where *ε* (length × time^−1^) is the denudation rate, *ρ* is rock density (mass × length^−3^), [OC_petro_] is the OC concentration in rock (mass × mass^−1^) and *χ* is the weathering intensity as the fraction of OC_petro_ weathered from rock. Weathering intensity *χ* has been shown to vary between low values of 0.2 in highly erosive settings^7^ and very high values of 0.98 in slow denudation settings^8^ with most falling in a range of 0.6–0.9 (refs. ^7,33,34^). Thus, *χ* presents a substantially smaller variance across environments in contrast to denudation rate and [OC_petro_], which vary spatially by more than four orders of magnitude.

To constrain the stock of OC_petro_ in the near surface, we use [OC_petro_] from the US Geological Survey rock geochemical database, combined with global lithological maps^13^ and spatial chemical lithology classifications^36^. Our geospatial model simulates a large global near-surface OC_petro_ stock, with the estimate and its interquartile range at ${1490}_{-980}^{+2580}$ Gt OC_petro_ in the first metre of bedrock. This estimate is consistent with a global estimate of 1,100 Gt OC_petro_ within the first metre of sedimentary rocks^14^, a reassessment of deep soil radiocarbon data which provides evidence for OC_petro_ inputs^20^, and is of comparable magnitude to that of global soils (2,060 ± 215 Gt OC)^37^ and marine sediments (2,322 ± 75 Gt OC)^38^. As opposed to soil OC stocks, the distribution of OC_petro_ is primarily controlled by the geological history of continents. While the highest [OC_petro_] is found in black shales (Extended Data Fig. 4), such rocks compose a tiny fraction of the Earth’s surface^13^, and instead, most OC_petro_ is found in fine-grained sedimentary deposits such as shales (Fig. 1b). Geospatial patterns reveal low OC_petro_ stocks on the African continent (Fig. 1b), owing to a low occurrence of fine-grained sedimentary rocks. In contrast, substantial portions of Eurasia, South America and the middle of North America east of the Rocky Mountains contain shales. The overlap of OC_petro_ stocks and patterns of denudation, driven mostly by rock uplift in mountains, determines the exposure of this OC stock to oxidative weathering. We estimated denudation using a probabilistic spatial model that incorporates catchment-scale cosmogenic radionuclide (CRN) denudation rates^39^, digital topography^40^ and lithological maps^13^. The resultant modelled global denudation rate and its interquartile range is ${11}_{-6}^{+13}$ Gt yr^−1^, within range of recent estimates of global denudation at ${28}_{-20}^{+64}$ Gt yr^−1^ (ref. ^16^) and 15 ± 2.8 Gt yr^−1^ (ref. ^17^).

## Spatial model of OC_petro_ oxidation

Rather than the more classical mean-field parametrization schemes previously employed to model OC_petro_ supply rates^14^, we use a probabilistic approach^41^ that accounts for the uncertainties in both variables in a spatial model. In each cell, empirical probability distributions of [OC_petro_] based on rock type (Extended Data Fig. 4) and probability distributions of denudation based on rock type and topographic slope (Extended Data Fig. 6) are sampled in 10,000 Monte Carlo simulations. We calibrated the geospatial model by minimizing the residuals between the modelled cell values of OC_petro_ oxidation rates (*J*_ox_) and our compilation of Re-proxy data at the river catchment scale (Methods). Thus, our approach describes the spatial patterns of oxidative weathering rate as a function of topographic slope and rock type, which leads to simulations that are consistent with an assessment of global rock nitrogen weathering patterns which are dominated by denudation of fine-grained sedimentary rocks^41^.

Using our spatial model, we estimate that oxidative weathering of OC_petro_ releases ${68}_{-6}^{+18}$ MtC yr^−1^ as CO_2_ from the land-surface environment. The flux is lower than our spatially uncorrected extrapolation of Re-proxy measurements (${98}_{-9}^{+28}$ MtC yr^−1^), consistent with the slight bias towards high denudation rate settings in the river basin dataset. The best estimate of the oxidative weathering flux is higher than an independent estimate of OC_petro_ erosion and river transport (that is, the export of OC_petro_ that has not been weathered) of ${43}_{-25}^{+61}$ MtC yr^−1^ based on river solid load composition and flux^19^, even though the uncertainties overlap. While a direct comparison of these estimates is difficult based on their quantification from dissolved versus particulate river chemistry and flux, they suggest an average weathering intensity of OC_petro_ of about 60%, which is consistent with studies from large river basins^19^ and intensities measured in soils^8,42^.

The OC_petro_ oxidation model can estimate the turnover time of OC_petro_ at the surface. When combined with OC_petro_ stocks, the model suggests that ${0.05}_{-0.03}^{+0.12}$% yr^−1^ of the global OC_petro_ stock in the first 10 cm of bedrock may be oxidized during denudation and weathering. A global OC_petro_ loss rate of about 0.05% yr^−1^ equates to a carbon turnover time (the ratio of total OC_petro_ to carbon outputs by oxidation) of approximately 2,000 years. This is about double the corresponding value for global soils^43^, but shorter than turnover times in tundras of approximately 3,900 years^44^. Given the large stock of OC_petro_ that we report (approximately 150 Pg C in the upper 10 cm) and its turnover time, OC_petro_ cannot be assumed to be inert and passive in the shallow subsurface. The input of OC_petro_ into soils can also impact soil residence time estimations and lead to an underestimation of soil carbon exchange fluxes with the atmosphere^20^.

Across the land surface, OC_petro_ weathering is relatively focused (Fig. 1d), with variations in rock type and relief, which drive OC_petro_ content and denudation, respectively, determining the magnitude of OC_petro_ oxidation and CO_2_ release. Large regions of the African continent have lower OC stocks in bedrock and have lower relief, which together limit OC weathering. In contrast, higher OC_petro_ oxidation rates are estimated for northern latitudes, where OC-rich rock and high-relief landscapes are more prevalent. Overall, 10% of the Earth surface with the highest OC_petro_ oxidation rates account for 60% of the global flux in our model. The world average rate is 0.5 tC km^−2^ yr^−1^, hotspots (surpassing ten times world average) and hyperactive areas (all areas surpassing five times world average) are responsible for 32% and 44% of CO_2_ emissions, respectively, while representing only 1.2% and 3% of ice-free terrestrial land area, respectively. OC_petro_ weathering rates in our model are more spatially concentrated than a 1-km resolution spatial model of silicate weathering^45^, where hotspots (0.51% by area) and hyperactive areas (2.9% by area) accounted for 8.6% and 19.6% of total CO_2_ consumption, respectively. This outcome appears reasonable because OC_petro_ is less common spatially than silicate minerals and reacts faster^3,25^.

## Weathering CO_2_ sources versus sinks

The OC_petro_ weathering flux and release of CO_2_ to the atmosphere of ${68}_{-6}^{+18}$ MtC yr^−1^ is similar to global terrestrial CO_2_ uptake by silicate weathering (94−143 MtC yr^−1^)^10^. Silicate weathering involves dissolved and gaseous CO_2_ uptake through bicarbonate production and the release of dissolved ions, some of which then precipitate as marine carbonate rocks^4^. The resultant total transfer of carbon from the atmosphere to the lithosphere by silicate weathering is 47−72 MtC yr^−1^. Besides their opposing impacts on the transfer of carbon between the atmosphere and lithosphere, fluxes of silicate weathering versus OC_petro_ oxidation may have contrasting responses to climate. Silicate weathering is invoked as negative feedback to climate warming through increased rates of silicate weathering from increased temperature and a more vigorous hydrological cycle, drawing down more CO_2_ (refs. ^35,46^). In contrast, in high denudation rate settings the CO_2_ release from OC_petro_ oxidation may increase with temperature^30,31^, while links to glacial erosion processes complicate the feedback between oxidative weathering and climate^33^.

**Fig. 2:**
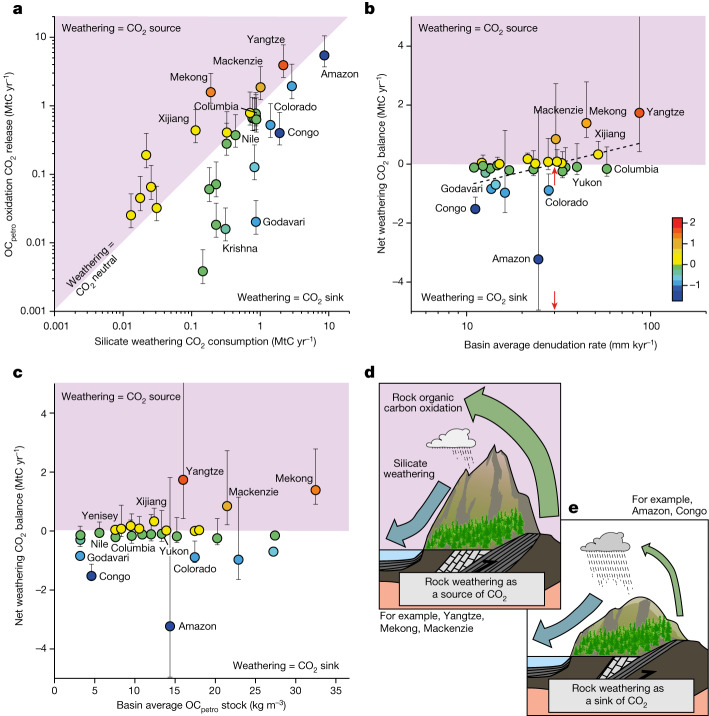
Earth’s major river basins, their silicate weathering carbon sinks and OC_petro_ weathering carbon sources, and their overall rock weathering budget based on these fluxes. **a**, Silicate^10^ versus OC_petro_ weathering fluxes and their net values. Basins that produce a net source of CO_2_ are shown in the shaded half of the plot, with the net magnitude of the weathering CO_2_ flux illustrated by the symbol colour (in MtC yr^−1^). **b**,**c**, Net weathering balance versus basin-average denudation (red arrow: cross-over at about 30 mm kyr^−1^) (**b**) and versus basin-average OC_petro_ stock (**c**). Error bars represent uncertainty of OC_petro_ oxidation model outputs based on the uncertainty of the training data (see Methods, ‘OC_petro_ oxidation yields and uncertainties’). **d**,**e**, Variable rates of uplift and erosion, climate and OC_petro_ stocks across Earth’s surface impact OC_petro_ and silicate weathering rates differently, leading to regions where rock weathering is a source (**d**) or a sink (**e**) of CO_2_. Source data

Silicate and OC_petro_ weathering processes may overlap, as sedimentary rocks contain silicate minerals as well as OC_petro_; however, the relative magnitude of these fluxes will vary spatially with climate, rock type and denudation^35,46^. We assess the net balance of rock weathering within major river basins (Fig. 2), using our OC_petro_ oxidation model and silicate weathering estimates^10^.

Within uncertainties, rock weathering in about a third of the major river basins is a net source of CO_2_ after OC_petro_ oxidation is considered, even while using the values of initial atmospheric CO_2_ consumption of silicate weathering rather than the smaller quantity of CO_2_ eventually locked up in the lithosphere through carbonate precipitation of the associated released dissolved ions (Fig. 2a). The Yangtze (Changjiang) and Mekong draining the eastern flanks of the Himalayas and the Mackenzie River draining shales west of the Rockies in Canada are major sources of CO_2_ from rock weathering. These high-emitting basins have in common some of the highest basin-average denudation rates and OC_petro_ stocks (Fig. 2b,c), which is consistent with OC_petro_ oxidation being driven by OC_petro_ stocks and denudation (equation (1))^7,8,25^.

Hotspots of CO_2_ release during rock weathering appear to lie at the edges of major active mountain ranges where relatively young, marine sedimentary deposits are uplifted and supplied to the oxidation process through denudation. Examples include the shales of the Himalayan collision zones and east of the Rocky Mountains (Fig. 1b,d). On the other hand, basins where rock weathering is the biggest net sink of CO_2_ do not necessarily lie at the extremes of low denudation and low OC_petro_ stocks. Though the tropical Congo River and volcanics-dominated Godavari River basins have low basin-average denudation rates and low OC_petro_ stocks, neither one is the biggest weathering sink; that distinction applies to the Amazon River basin, which lies in the global middle range of denudation rates and OC_petro_ stocks (Fig. 2b,c). There, the kinetically limited silicate weathering reaction benefits from long sediment residence times and a warm, humid climate.

While the Andes is a hotspot for OC_petro_ oxidation fluxes (Fig. 1d), the exceptionally large lowland drainage area of the Amazon means that OC_petro_ oxidation may be supply limited. In a third of river basins weathering remains carbon neutral within uncertainty; for example, such is the case with the volcanic-rich Columbia River catchment.

**Fig. 3:**
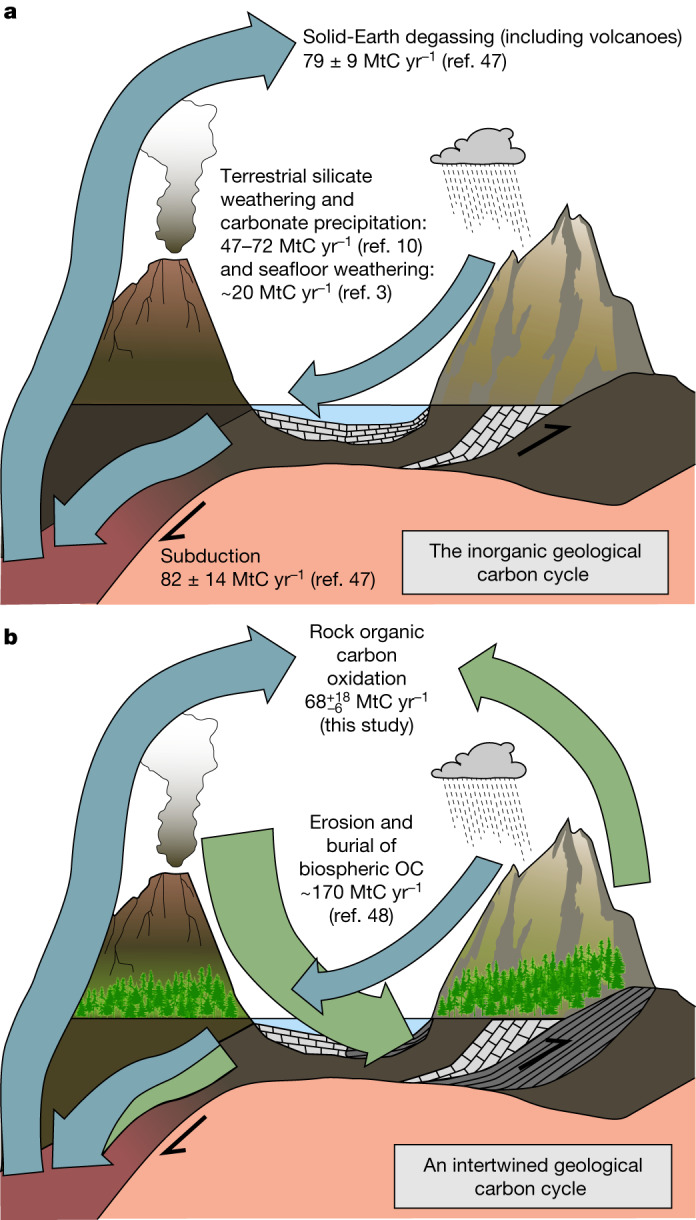
A shift in understanding the geological carbon cycle. **a**, The inorganic geological carbon cycle relies on a global balance between solid-Earth CO_2_ degassing and silicate weathering. **b**, The emerging understanding of the role of organic carbon in the global geological carbon cycle, supported by the high flux of OC_petro_ oxidation reported in this study. Hence, the biological, chemical and physical processes of biospheric OC production, burial and release control long-term climate variability and stability.

To avoid large swings in atmospheric CO_2_ over millions of years and maintain an apparent close balance of CO_2_ sources and sinks^2,11^, any potential imbalances in weathering-derived carbon fluxes must be addressed by accounting for other components in the long-term carbon cycle. Solid-Earth degassing associated with volcanoes and diffuse release from metamorphism in subduction zones is responsible for 79 ± 9 MtC yr^−1^ released into the atmosphere (Fig. 3a)^47^, while any additional (non-subduction) global CO_2_ release during orogenic metamorphism and sulfide oxidation and inorganic C uptake during seafloor weathering are more poorly constrained^3^. As our results show that the weathering of OC_petro_ offsets silicate weathering in the long-term carbon cycle, a large additional sink of CO_2_ is needed. This may be provided by burial of organic matter in ocean sediments, which could contribute as much as 170 MtC yr^−1^ (Fig. 3b)^48^. In addition, as OC_petro_ fluxes can overtake silicate weathering during periods of more intense uplift and erosion (Fig. 2b,d and Extended Data Fig. 8), the question whether orogenic periods in Earth history are sources or sinks of atmospheric CO_2_ is now a reopened question^3,31,49,50^. The net balance will depend on factors such as transport of terrestrial biospheric carbon to oceans (${157}_{-50}^{+74}$ MtC yr^−1^) and its burial^19^. A global comparison of catchment-scale OC_petro_ oxidation yields and estimated terrestrial biospheric OC burial (Extended Data Fig. 8) suggest the OC burial can apparently offset or even overcompensate CO_2_ release from OC_petro_ oxidation. This understanding persists when the additional marine OC burial sink in sediment is factored into global flux estimates (Fig. 3b)^48^. The dynamics of Earth’s weathering thermostat thus need to be revisited to account for variation in all these fluxes and consider how their relative importance may have changed as life evolved and the OC stocks of sedimentary rocks have increased^3,22^.

## Methods

**Extended Data Fig. 1:**
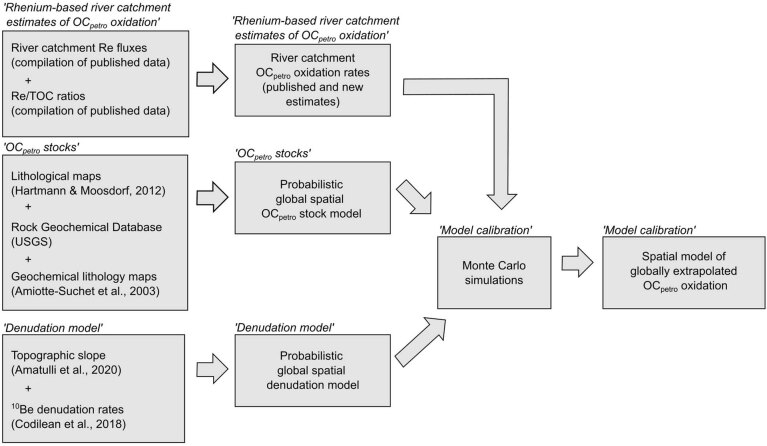
Flow chart of data, subroutines and outcomes and the relevant Methods sections. Probabilistic geospatial models of OC_petro_ stocks and denudation are used to extrapolate a global compilation of river catchment OC_petro_ oxidation rates across the Earth’s surface.

The workflow of materials and methods (Extended Data Fig. 1) starts with the compilation and derivation of rhenium-concentration-based OC_petro_ oxidation flux estimates, discussed just below. Then we detail the ‘Global spatial OC_petro_ oxidation model’ and its application of submodels for OC_petro_ stocks and denudation, and the Monte Carlo routines used in the model’s approach. Finally, we discuss the ‘Limitations and uncertainties’ involved in our methods and calculations.

### Rhenium-based river catchment estimates of OC_petro_ oxidation

From a series of dissolved rhenium measurements (typically completed by ICP-MS), the dissolved Re flux *J*_Re_ (t yr^−1^) can be used to estimate OC_petro_ oxidation flux, *J*_OCpetro-ox_ (tC yr^−1^) using:

2 $${J}_{{\rm{O}}{\rm{C}}{\rm{p}}{\rm{e}}{\rm{t}}{\rm{r}}{\rm{o}}-{\rm{o}}{\rm{x}}}=\,{J}_{{\rm{R}}{\rm{e}}}\times {\left(\frac{[{{\rm{O}}{\rm{C}}}_{{\rm{p}}{\rm{e}}{\rm{t}}{\rm{r}}{\rm{o}}}]}{[{\rm{R}}{\rm{e}}]}\right)}_{i}\times {f}_{{\rm{c}}}$$

where *f*_c_ is the fraction of dissolved Re derived from OC_petro_ oxidation^34^ and ([OC_petro_]/[Re])_*i*_ is the organic carbon to rhenium concentration ratio (g g^−1^) in rocks of type *i* undergoing weathering. In some catchments where it may be important, an additional term, not shown in equation (2), has been applied to correct for the presence of graphite, which may not undergo alteration during weathering^33^.

#### Compiled published measurements

In this study we compile estimates of OC_petro_ oxidation using the dissolved Re proxy from published literature (Supplementary Table 1). These include the Yamuna River, India^24^; ten Taiwanese rivers^7^; four rivers from the western Southern Alps^33^; four rivers from the Mackenzie Basin, Canada^3,34^; and six rivers draining the Peruvian Andes^51^. Two Swiss catchments^25^ are not included because of their very small catchment area compared to the geospatial scales over which we complete the upscaling.

For some of these case studies, dissolved rhenium flux has been estimated from repeated sampling and discharge records^34^, while earlier studies all include single snapshot samples^7,24^, and all include measurements of the local sedimentary rock composition. Most of these compiled studies have used dissolved ion ratios to estimate the source of dissolved Re, akin to *f*_c_ (equation (2)), apart from the Taiwan dataset^7^. While uncertainties on the OC_petro_ oxidation yields appear relatively large (Supplementary Table 1), it is important to note that the measured range in yields is much larger than the uncertainties.

#### New estimates of OC_petro_ oxidation

To expand the 25 estimates of OC_petro_ oxidation from river catchments described previously, we build on a previous study of dissolved Re fluxes in large rivers that reports dissolved Re concentrations and fluxes for major basins around the world^9^. We use these measurements and combine them with estimates of *f*_c_ and ([OC_petro_]/[Re])_*i*_, discussed in the following sections, to calculate OC_petro_ oxidation yields with associated uncertainties using published approaches^25,34^.

In locations with substantial local sources of fossil fuel combustion (for example, coal-fired power plants or steel works), rainwater can contain concentrations of Re that approach those of river water^8,52^, whereas locations that have minimal impacts from local pollution sources have Re concentrations in rainwater that are below detection^25,33^. In the large river dataset^9^, some large rivers are noted to have markedly increased Re concentrations and fluxes; the conclusion is that this was due to anthropogenic Re inputs. In a first-order catchment of the Mississippi Basin, this has been confirmed by a detailed Re mass balance^52^. A study of Re across Indian catchments suggests that while Re in Himalayan catchments and the mainstem Ganges and Brahmaputra behave conservatively, peninsular lower relief catchments with denser populations and industrial activity suggest anthropogenic inputs^53^. For this study’s purpose, to quantify weathering reactions, we only use Himalayan rivers and the mainstem Ganges and Brahmaputra in India, and we have further excluded Re data from the Danube, Mississippi and Yangtze rivers from our analysis. Our addition of catchment Re data to the Miller dataset includes a large contribution of small upland catchments with higher average erosion rates, where the authors of these studies selected sites with minimal human disturbance (Supplementary Table 1). We further consider the role of anthropogenic Re in our model results in the ‘Limitations and uncertainties’ section.

#### Estimation of Re source and *f*_c_

To estimate the fraction *f*_c_ of dissolved Re sourced from OC_petro_ for the rivers in the Re flux dataset^9^, we follow a previously used forward model mixing approach^25,34^:

3 $${{\rm{Re}}}_{{\rm{OC}}}={{\rm{Re}}}_{{\rm{tot}}}\times {f}_{{\rm{c}}}={{\rm{Re}}}_{{\rm{tot}}}-{{\rm{Re}}}_{{\rm{sulf}}}-{{\rm{Re}}}_{{\rm{sil}}}$$

where Re_OC_ is the rhenium concentration of OC_petro_-derived Re in the dissolved load, Re_tot_ is the measured Re concentration, Re_sulf_ and Re_sil_ are the concentrations derived from weathering of sulfide and silicate minerals, respectively. These unknowns can be quantified as:

4 $${{\rm{Re}}}_{{\rm{sulf}}}=\left[{{\rm{SO}}}_{4}\right]\times {\left(\frac{\left[{\rm{Re}}\right]}{\left[{{\rm{SO}}}_{4}\right]}\right)}_{{\rm{sulf}}}$$

5 $${{\rm{Re}}}_{{\rm{sil}}}=\left[{\rm{Na}}\right]\times {\left(\frac{\left[{\rm{Re}}\right]}{\left[{\rm{Na}}\right]}\right)}_{{\rm{sil}}}$$

where the element ratios of the end members for silicate, ${\left(\left[{\rm{Re}}\right]/\left[{\rm{Na}}\right]\right)}_{{\rm{sil}}}$, and sulfide, ${\left(\left[{\rm{Re}}\right]/\left[{{\rm{SO}}}_{4}\right]\right)}_{{\rm{sulf}}}$, are defined, and with the assumption that the dissolved sulfate (SO_4_) and sodium (Na) respectively derive only from sulfide oxidation and silicate weathering and are conservative. This returns an upper bound on the Re_sulf_ and Re_sil_ components (Supplementary Table 2). Following recent work^34^, we use a range of values for each, where ${\left(\left[{\rm{Re}}\right]/\left[{{\rm{SO}}}_{4}\right]\right)}_{{\rm{sulf}}}$ ranges from 0.2 × 10^−3^ to 4 × 10^−3^ pmol µmol^−1^ (ref. ^23^) and ${\left(\left[{\rm{Re}}\right]/\left[{\rm{Na}}\right]\right)}_{{\rm{sil}}}$ ranges from 0.4 × 10^−3^ to 2 × 10^−3^ pmol µmol^−1^. Here, we correct Na^+^ concentrations for atmospheric-derived Na, [Na^+^]*, where [Na^+^]* = [Na^+^] − [Cl^−^] × 0.8, assuming all Cl^−^ derives from precipitation, which has a molar [Na^+^]/[Cl^−^] ratio of 0.8. We similarly correct for atmospheric SO_4_ inputs.

#### Constraints on ([OC_petro_]/[Re])_*i*_

**Extended Data Fig. 2:**
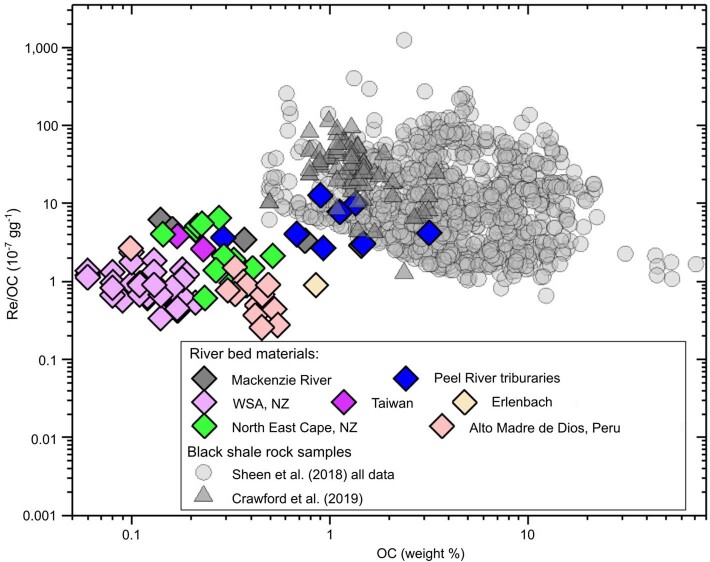
Rhenium (Re) to organic carbon (OC) concentration ratio (10^−7^ g g^−1^) in published black shales^54,67^ alongside those measured in riverbed materials (Supplementary Table 3). Peel riverbed materials capture the input of OC from black shale lithologies, which in other basins are much less common.

A recent compilation^54^ provides measurements of ([OC_petro_]/[Re])_i_ from rock samples of different ages around the world. However, most of these measurements were made on black shales with OC_petro_ contents greater than 1%, which occur on only 0.3% of the Earth surface (Extended Data Fig. 5). Riverbed material sediments from erosive catchments provide an alternative way to capture landscape-scale average rock composition, albeit with some potential for weathering to alter the primary signal. Here we compile measurements of [OC_petro_]/[Re] on bed materials from rivers around the world (Supplementary Table 3 and Extended Data Fig. 2) and supplement this dataset with additional samples from mudrocks of the Eastern Cape, New Zealand, and the Peruvian Andes measured using methods described previously^25^. We find that regions with lower OC_petro_ concentrations that are more typical of sedimentary rocks at the continental surface—units including fine-grained sedimentary rocks that make up more than 35% of Earth surface (Extended Data Fig. 5)—have lower and more consistent ratios of OC and Re in their rocks. The samples from the Peel River in the Mackenzie River basin^34^ overlap the lower end of the published black shale values. Since this is the catchment with the highest proportion of black shales in our Re dataset, these samples allow us to capture the imprint of this important marginal lithology at the landscape scale.

**Extended Data Fig. 3:**
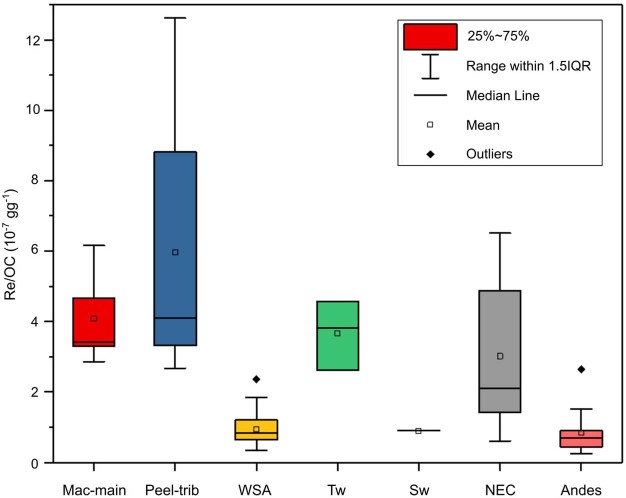
Rhenium (Re) to organic carbon (OC) concentration ratio (10^−7^ g g^−1^) of riverbed materials, organized by sample location (Supplementary Table 3). Conservatively, the Monte Carlo analysis of OC_petro_ oxidation rates from the dissolved rhenium proxy uniformly samples the complete measured range of Re/OC values presented here. Sample groups are as follows: Mac-main = Mackenzie River; Peel-trib = Peel river tributaries; WSA = Western Southern Alps, New Zealand; Tw = Taiwan rivers; Sw = Swiss rivers; NEC = North East Cape, New Zealand; Andes = Madre de Dios catchment.

The bedrock composition in the catchments of rivers studied in the Re flux dataset^9^ is not reported. However, we note the good geographic coverage and number of samples that we have from riverbed materials from erosive settings around the world. These provide constraint on the initial OC to rhenium ratio in the rocks. To conservatively quantify uncertainty in the range of OC_petro_ oxidation rates from dissolved-Re data, we perform a Monte Carlo simulation in which we uniformly sample the entire range of measured [OC_petro_]/[Re] values, from low values indicative of carbon-poor and/or metamorphic rocks 2.5 × 10^−8^ g g^−1^ (ref. ^33^) towards 1.26 × 10^−6^ g g^−1^ (ref. ^34^) in catchments with higher OC in rocks (Supplementary Table 3 and Extended Data Fig. 3).

#### OC_petro_ oxidation yields and uncertainties

Equation (2) is used for each basin in the Re flux dataset^9^. Uncertainties in *f*_c_ derive from the range of values used in the sulfide and silicate end member compositions (equations (4) and (5)). For [OC_petro_]/[Re], we use the range of values discussed in the just-previous section on constraints (Extended Data Fig. 3). A Monte Carlo uncertainty propagation is used on these variables, with 10,000 randomly selected combinations of input values (with uniform sampling) are used to estimate *J*_OCpetro-ox_ for each basin. The median value of the Monte Carlo simulation and the interquartile range are reported (Supplementary Table 1).

#### Geospatial catchment boundaries

To derive the catchment outlines and areas corresponding to the Re-proxy samples in our compiled dataset, we used the HYDROSHEDS flow direction grid at 3 arc-second resolution^55^ and ArcGIS Pro^56^. Catchments outside the latitudinal cover of HYDROSHEDS were derived from the HYDRO1K flow direction grid^57^ and catchments in Iceland were derived from ALOS AW3D using TauDEM functionality in OpenTopography^58^. While most published sample coordinates (Supplementary Table 1) give the correct location on the cited drainage systems, in a handful of cases, coordinates had to be amended by up to a few kilometres, which may reflect errors in transcribing (for example, Kikori and Purari^9^). Final quality control included a comparison of the extracted drainage basin areas and those published, with good agreement overall (less than 2% residual). However, some drainage areas cited in the Re flux dataset^9^ refer to the river mouth, rather than the river catchment upstream of the Re sample location. In these cases, we use the Re sample location and its upstream catchment. Finalized coordinates of Re samples determined for each drainage system, with the corresponding upstream drainage area, are given in Supplementary Table 1. Spatial files of upstream drainage boundaries and Re sample locations are available on Zenodo (available from 10.5281/zenodo.8144244). To convert dissolved Re concentrations into Re fluxes, average annual water discharge was calculated using published numbers at gauges (Supplementary Table 1) and scaled to the upstream drainage area of Re sample locations.

In addition to spatial catchment boundaries for the Re proxy dataset, we compare our spatial model output to published estimates of silicate weathering^10^ that use the GRDC dataset in *Major River Basins of the World*^59^. Drainage areas used by ref. ^10^ have slight discrepancies with those found in the GRDC dataset. We account for these in our analysis of major river basin net weathering flux (Supplementary Table 4).

### Global spatial OC_petro_ oxidation model

In the following three sections, we provide additional rationale and details of the modelling approaches. The model procedures apply two spatial probabilistic subroutines; one deals with OC_petro_ stocks in surface rocks and the other with spatially defined denudation rates. These are combined in a Monte Carlo framework alongside the Re-proxy river catchment data to optimize the model and then extrapolate OC_petro_ oxidation rates (Extended Data Fig. 1). Model simulations were implemented at 1-km grid scale (Mollweide projection, WGS84 datum) in the Python programming language^60^.

#### OC_petro_ stocks

**Extended Data Fig. 4:**
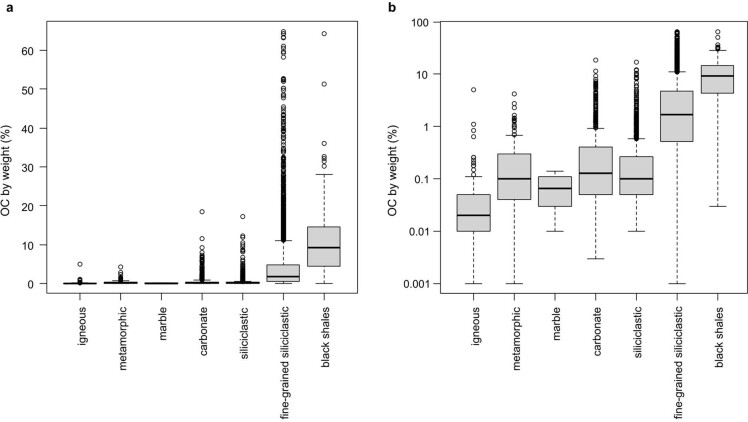
Organic carbon concentrations (OC in weight %) of lithological types in the USGS Rock Geochemical Database (a) and the same figure in log scale (b). Black lines show median values, boxes show the interquartile range (IQR), whiskers show IQR + 1.5 × IQR, and outliers are shown as open symbols (although still included in the analysis). Source data

**Extended Data Fig. 5:**
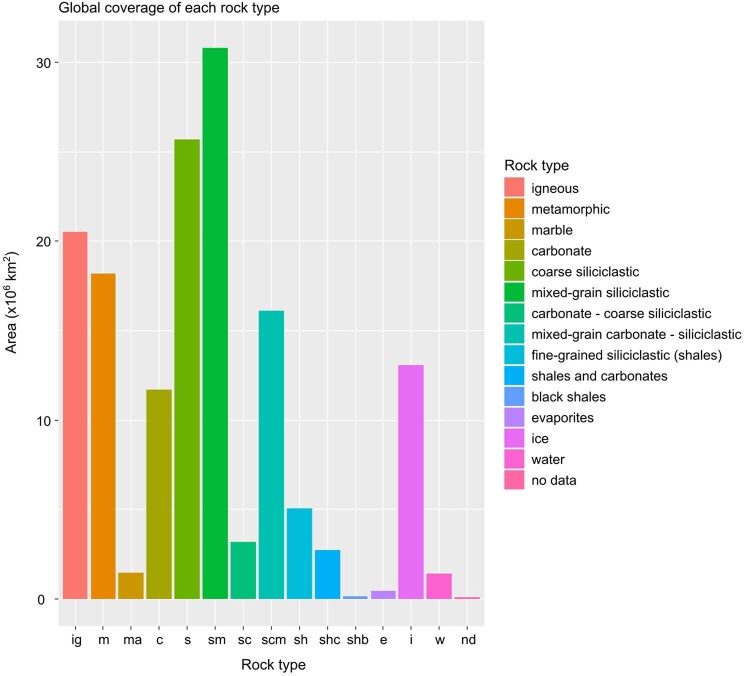
Total continental area (m^2^) of each mapped units from the GLiM model^13^. Mapped units include mixed units of siliciclastic lithologies with various grain-sizes (fine and coarse) and mixes of carbonate and siliciclastic lithologies^13^. Source data

Rock samples from the USGS Rock Geochemical Database, sorted into lithological categories (Supplementary Table 5), were mapped onto units of the highest-resolution global lithological maps currently available^13^. Extended Data Fig. 4 shows the OC_petro_ concentration of key lithologies in the USGS Rock Geochemical Database. Weight percentage values from the USGS Rock Geochemical Database were converted to OC_petro_ stock using rock densities (Supplementary Table 5). In our Monte Carlo framework, OC_petro_ stocks at each grid cell were sampled independently using the empirical distributions of rock OC_petro_ content derived from both the USGS database (Extended Data Fig. 4) and our unit classification (Supplementary Table 5). In our lithology model, complex mapped units present in GLiM consist of a combination of carbonates and silicates of various grain sizes (Extended Data Fig. 5 and Supplementary Table 5). To calculate the OC_petro_ reservoir among these units, we derive the fractional abundance of lithology types (*F*_*n*_) from continental-scale literature estimates^36^:

6 $${[{{\rm{O}}{\rm{C}}}_{{\rm{p}}{\rm{e}}{\rm{t}}{\rm{r}}{\rm{o}}}]}_{{\rm{r}}{\rm{o}}{\rm{c}}{\rm{k}}}={F}_{1}({[{{\rm{O}}{\rm{C}}}_{{\rm{p}}{\rm{e}}{\rm{t}}{\rm{r}}{\rm{o}}}]}_{{\rm{l}}{\rm{i}}{\rm{t}}{\rm{h}}{\rm{o}}{\rm{l}}{\rm{o}}{\rm{g}}{\rm{y}},1})+{F}_{2}({[{{\rm{O}}{\rm{C}}}_{{\rm{p}}{\rm{e}}{\rm{t}}{\rm{r}}{\rm{o}}}]}_{{\rm{l}}{\rm{i}}{\rm{t}}{\rm{h}}{\rm{o}}{\rm{l}}{\rm{o}}{\rm{g}}{\rm{y}},2})+\,\cdots \,+{F}_{n}({[{{\rm{O}}{\rm{C}}}_{{\rm{p}}{\rm{e}}{\rm{t}}{\rm{r}}{\rm{o}}}]}_{{\rm{l}}{\rm{i}}{\rm{t}}{\rm{h}}{\rm{o}}{\rm{l}}{\rm{o}}{\rm{g}}{\rm{y}},n})$$

7 $${F}_{1}+{F}_{2}+{\rm{\cdots }}+{F}_{n}=1$$

#### Denudation model

**Extended Data Fig. 6:**
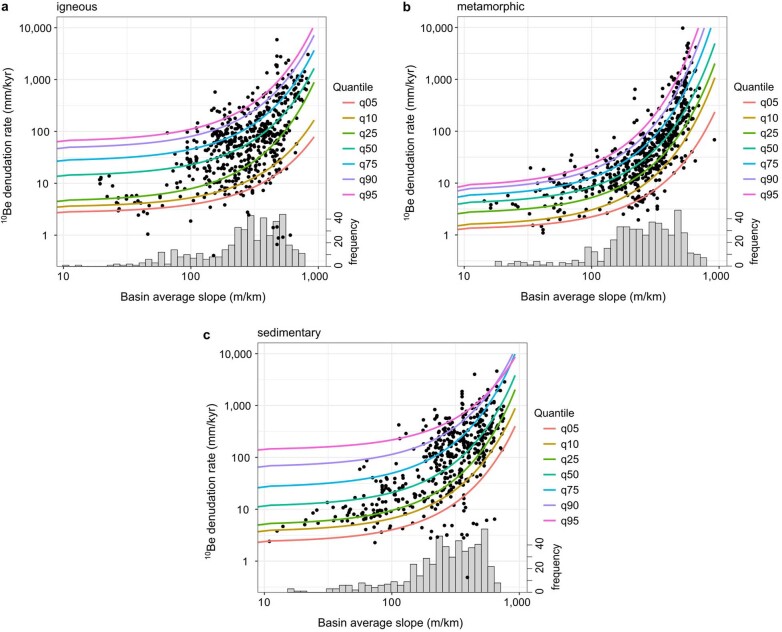
Cosmogenic isotope-derived denudation rates (^10^Be erosion rate mm/kyr) from the OCTOPUS dataset^39^ analysed here. Sites are grouped by dominant lithology and the denudation rate as a function of basin average slope (m/km) returned by quantile regression for (**a**) igneous, (**b**) metamorphic, and (**c**) sedimentary dominated catchments. The quantiles corresponding to each grid cell slope value constitute cumulative density functions (CDFs) which can be sampled in each Monte Carlo simulation that quantifies global oxidative weathering rates (Extended Data Fig. 1). Source data

The denudation model is parametrized using a regression approach, similar to techniques applied elsewhere^16,41^. We regressed a compilation of long-term catchment-scale ^10^Be denudation estimates^39^ against mean local slope generated from the Geomorpho90m dataset^40^. Mean local slope was calculated using the focal statistics tool in ArcGIS Pro^56^ and the Geomorpho90m slope dataset with a 5-km moving radius. Slope values were then matched to ^10^Be denudation estimates at a single cell based on the reported longitude and latitude. A quantile regression approach^41,61,62^, allows us to mitigate over- and underestimations inherent in using a mean model fit to the global land surface^16^ (Extended Data Fig. 6). For each unique slope value in the global raster, denudation quantiles were used to construct a cumulative distribution function which could be sampled in each Monte Carlo run (compare ref. ^41^).

We account for differential erodibilities of sedimentary, crystalline metamorphic and igneous rock types by running regressions between slope values and ^10^Be values for each rock type (Extended Data Fig. 6). Thus, only ^10^Be values from catchments dominated (more than 80%) by one rock type are used in this regression. This accounting of erodibilities is important, as OC-rich shales are weaker and more erodible than OC-poor strong igneous rocks. Residuals between the CRN denudation dataset and the modelled denudation do not change when differential rock erodibility is considered. However, when combined with our OC_petro_ stock model, the rock erodibility-corrected OC supply rate model results in 20% higher rates. We also consider the grid-scale bias considered by previous workers^16,41^: as DEM resolution decreases, slope—as the spatial derivative of elevation—decreases, resulting in an artificial flattening effect^16^. As our Monte Carlo framework is computationally intensive, using a 90-m-resolution global raster input would not be feasible. However, we use a 90-m-resolution slope dataset to run regression curves as shown in Extended Data Fig. 6, after which we output a 90-m-resolution raster dataset of estimated denudation rates using the median regression curve. By resampling the raster dataset of estimated denudation rates to 1-km resolution after conversion from slope values, we avoid the bias that can lead to an underestimation of denudation by the flattening effect. In our Monte Carlo framework, the quantile regression curves for each raster value can then be sampled to draw a representative denudation value out of the empirical distribution of denudation rates.

#### Model calibration

**Extended Data Fig. 7:**
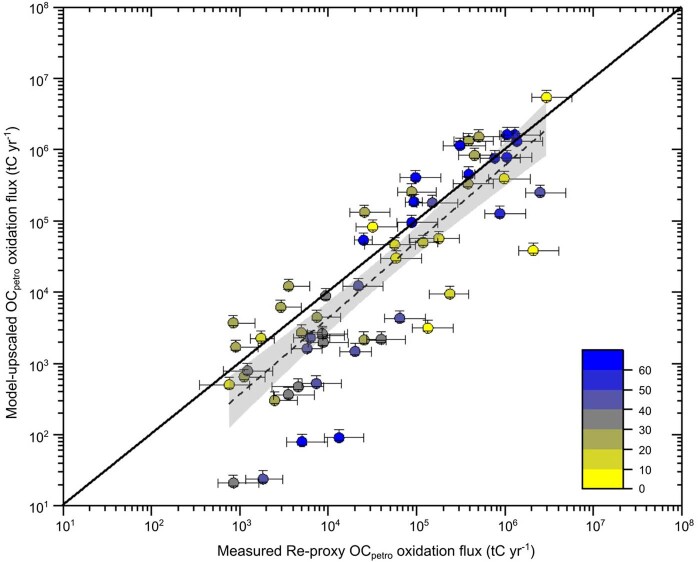
Rock organic carbon (OC_petro_) oxidation rate (tC yr^−1^) from the Re-proxy versus the model upscaled output for the global set of catchments used in this study (Supplementary Table 1). Catchments are coloured by the latitude of the sampling point (0° to 80° N and S). Error bars represent uncertainty in Re-proxy values for OC_petro_ oxidation (see Methods sections headed ‘Rhenium-based river catchment estimates of OC_petro_ oxidation’ & ‘OC_petro_ oxidation yields and uncertainties’) and the resultant uncertainty in extrapolation model outputs. The dashed line and grey shading shows fit to the data (95% confidence intervals) and the solid black line shows 1:1 line.

**Extended Data Fig. 8:**
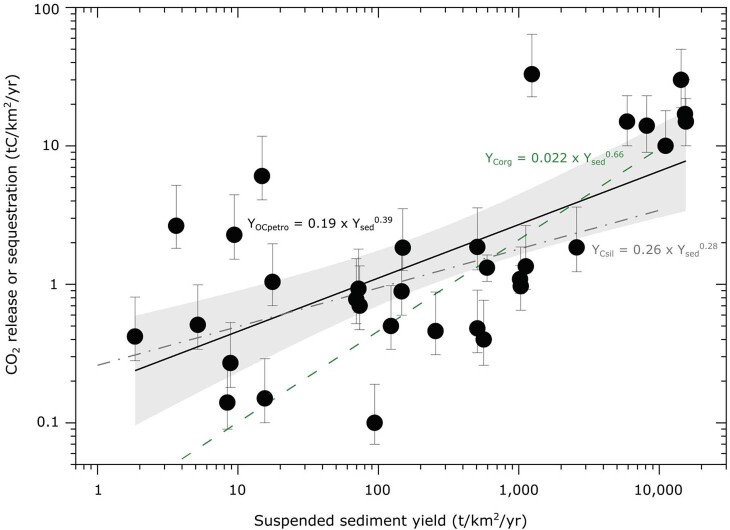
CO_2_ release through OC_petro_ oxidation (black dots, this study; Y_OCpetro_, black line) is more sensitive to sediment yield than CO_2_ sequestration through silicate weathering (Y_Csil_, grey dotted-dashed line)^4^, whilst terrestrial biospheric OC burial (Y_Corg_, green dashed line)^19^ could be even more sensitive. CO_2_ sequestration trendlines are from Galy et al.^19^ The black dots show CO_2_ release through OC_petro_ oxidation for all the Re sample catchments which have suspended sediment yield data available from the Land2Sea database^68^ (Supplementary Table 1) to allow comparison with the previous assessment of silicate weathering vs biospheric OC burial^19^. The exponent for Y_OCpetro_ is 0.39 ± 0.08 with r^2^ = 0.43, reflecting the scatter in data owing to the spatial dependence of OC_petro_ oxidation on rock OC content and denudation rates (Fig. 2). Therefore, comparisons based on the global trends in this plot, without considering their spatial context (see Fig. 2 for spatially explicit comparisons), should be done with care considering the low certainty.

The global model is calibrated by minimizing the residual with the Re-proxy-based estimates of OC_petro_ oxidation (tC yr^−1^) from 59 globally distributed river basins (Supplementary Table 1). Model selection was performed by running a Monte Carlo simulation (10,000 runs), using the variable OC_petro_ stock and denudation models described above, to find the output which minimizes total residuals across all 59 calibration basins simultaneously, such that the sum of all basin residuals was less than 1%. These simulations were run on the University of Oxford’s Advanced Research Computing (ARC) facility, taking about 24 core hours per simulation. The residuals of individual basins can be quite large for the biggest catchments (for example, the Amazon basin), but the relative residual, especially for the larger basins, falls within the uncertainty of model outputs, while accurately predicting the total OC_petro_ oxidation flux globally (Extended Data Fig. 7). We note that, overall, in basins with moderate OC_petro_ oxidation fluxes, the model may return conservative estimates. However, because this model has the advantage of being globally and spatially explicit, regional over- and underestimation of OC_petro_ oxidation found mostly at a local scale (less than 10,000 km^2^) tend to trade off while we are able to capture larger regional differences due to tectonics and geological setting (Fig. 1d).

### Limitations and uncertainties

There is a temporal mismatch between the CRN denudation data that inform our probabilistic denudation model, and our Re-proxy calibration data. The Re-proxy-based OC_petro_ oxidation fluxes used to calibrate our spatial extrapolation model capture fluxes from global rivers within the past decade or less. The CRN technique integrates denudation fluxes that span a millennium or more. Anthropogenic land-use change has doubled erosion and weathering since the early 1900s (ref. ^63^); hence, our global scale estimates of OC_petro_ oxidation rates reflect the combined influence of natural and anthropogenic activities on global weathering rates, which cannot be deconvolved in this present study.

Results of model versus Re-predicted OC_petro_ oxidation fluxes help us assess the potential for anthropogenic Re input to impact our estimates (Extended Data Fig. 7). We have considered anthropogenic Re inputs by removing three large river basins from a previous compilation^9^ and by adding carefully selected river catchment sites to our Re dataset (see the Methods section headed ‘Rhenium-based river catchment estimates of OC_petro_ oxidation’). In addition, our conversion of Re fluxes to OC_petro_ oxidation is conservative because we uniformly sample the range of Re/OC ratios starting at the lowest measured Re/OC ratio (see the Methods section headed ‘Constraints on ([OC_petro_]/[Re])’). This leads to error bars within our estimates that are conservatively large. Most notably, the model outputs of OC_petro_ oxidation versus the Re-estimated fluxes for each basin (Extended Data Fig. 7) show a tendency for the model to underpredict smaller catchments more than larger catchments. Our confidence in the weathering signal from Re in the small upland catchments is highest, and the upland, high-erosion-rate regions that these catchments sample contribute a dominant proportion of the global OC_petro_ flux in our model. While we cannot completely deconvolve the effect of anthropogenic Re in our constraints, we have confidence that the effect is unlikely to result in a significant overprediction of global OC_petro_ flux estimates.

The global extrapolation of OC_petro_ oxidation proxy data attempts to account for the dataset’s underlying heterogeneities in denudation and OC_petro_ stocks. However, it does not consider variability in temperature or precipitation which may control weathering—as seen in small-scale field measurements of OC_petro_ oxidation at sites of high denudation^30,31^. This is primarily due to the size of the Re-proxy catchment database, its spatial coverage and uncertainties inherent in any proxy approach. While the Re-proxy dataset is latitudinally variable (Fig. 1a) the model misfit minimization procedure shows the first-order controls on flux by OC_petro_ stock and denudation (Extended Data Fig. 7), meaning that any climatic controls on weathering could be not resolved at the global scale. We note that any bias introduced by extrapolating the global Re-proxy data without including climatic spatial controls on weathering intensity is likely to be minimal, because the underlying dataset spans from the tropics to Arctic locations.

Previous work has suggested that OC_petro_ oxidation and cycling of other OC pools can take place during floodplain transport in large fluvial systems^64,65^. While the Re-proxy dataset includes large basins with extensive floodplain areas (Fig. 1a), our model–data misfit approach may attribute the downstream fluxes to any higher denudation parts of the catchment. When the model is then upscaled, in lowland floodplain areas where fluvial processing and recycling of sediment^65^ can allow biogeochemical reactions to continue^65^, we may predict conservative OC_petro_ oxidation rates. At present, we lack empirical data to partition weathering between mountain and floodplain sections^3^. An alternative way to view this is that the removal of alluvial domains from contributing to denudation (since these are depositional) holds minimal control on the overall estimate (less than 1%). This result comes from a comparison of model outputs under two parametrization schemes: one where denudation occurs over all ice-free lands versus one where denudation only occurs in ice-free non-alluvial landscapes. Overall, the model’s largest contributor to uncertainty is in the conversion of dissolved Re fluxes to OC_petro_ oxidation estimates, which are extrapolated in our spatial model. This conversion depends on [OC_petro_]/[Re] ratios which introduce most of the uncertainty in the resulting OC_petro_ oxidation rates (see the Methods section ‘OC_petro_ oxidation yields and uncertainties’) and therefore in the model’s global output. More constraints on the relative ‘grey shale’ versus ‘black shale’ contribution to catchment Re fluxes could help tighten uncertainty in [OC_petro_]/[Re] ratios (see ref. ^66^ for a discussion).

Finally, our model includes implicit assumptions and features of the datasets which must be acknowledged. First, the model assumes a steady state, which might not accurately describe OC_petro_ oxidation in regions responding to changes in uplift, deglaciation or human activities, which may not yet have reached steady-state conditions.

Second, most catchment-scale CRN denudation data used in our model derive from lithologies that are quartz-rich and coarse-grained. These typically have lower erodibilities, potentially leading to an underestimation of denudation rates of softer shales which contain the majority of OC_petro_ stocks.

## Online content

Any methods, additional references, Nature Portfolio reporting summaries, source data, extended data, supplementary information, acknowledgements, peer review information; details of author contributions and competing interests; and statements of data and code availability are available at 10.1038/s41586-023-06581-9.

### Supplementary information

Peer Review File

Supplementary Tables 1–5 Table 1 includes Re sample data, catchment locations and other data used to calculate dissolved rhenium-derived OC_petro_ oxidation fluxes. Table 2 includes Re concentration data from the Re flux dataset^9^ used in this study. Table 3 includes data on riverbed material and its OC and Re concentrations. Table 4 includes outputs of global river silicate weathering^10^ compared with our OC_petro_ oxidation model outputs. Table 5 contains the rock type classification for mapping characteristic OC_petro_ stocks.

### Source data

Source Data Fig. 2

Source Data Extended Data Fig. 4

Source Data Extended Data Fig. 5

Source Data Extended Data Fig. 6

## Data Availability

All dissolved rhenium sample data are available in Supplementary Tables 1–5, in addition to which geospatial data, including those for Fig. 1, are deposited in a Zenodo repository (10.5281/zenodo.8144244). Source data are provided with this paper.

## Code availability

The spatial OC_petro_ oxidation model contains two spatial probabilistic subroutines of (1) OC_petro_ stocks in surface rocks and (2) spatially defined denudation rates. These are combined in a Monte Carlo framework alongside the Re-proxy river catchment data to optimize the model and then extrapolate OC_petro_ oxidation rates (Extended Data Fig. 1). Model simulations were implemented at 1-km grid scale (Mollweide projection, WGS84 datum) in the Python programming language. R and Python code, their environments and the necessary data files to run these are all deposited in a Zenodo depository (10.5281/zenodo.8144244) and additionally code is available from GitHub: https://github.com/jessezondervan/Global_OCpetro_Oxidation.
